# Distinguishing post-treatment changes from recurrent disease in cholangiocarcinoma: a case report

**DOI:** 10.1186/1752-1947-2-76

**Published:** 2008-03-07

**Authors:** Timothy N Showalter, A Omer Nawaz, Frederick M Fellin, Pramila R Anne, Ernest L Rosato, Adam P Dicker

**Affiliations:** 1 Department of Radiation Oncology, Thomas Jefferson University, Philadelphia, Pennsylvania, USA; 2 Department of Medical Oncology, Thomas Jefferson University, Philadelphia, Pennsylvania, USA; 3 Department of Surgery, Thomas Jefferson University, Philadelphia, Pennsylvania, USA

## Abstract

**Introduction:**

Three-dimensional techniques for radiotherapy have expanded possibilities for partial volume liver radiotherapy. Characteristic, transient radiographic changes can occur in the absence of clinical radiation-induced liver disease after hepatic radiotherapy and must be distinguished from local recurrence.

**Case presentation:**

In this report, we describe computed tomography changes after chemoradiotherapy for cholangiocarcinoma as an example of collaboration to determine the clinical significance of the radiographic finding.

**Conclusion:**

Because of improved three-dimensional, conformal radiotherapy techniques, consultation across disciplines may be necessary to interpret post-treatment imaging findings.

## Introduction

Conformal radiotherapy (RT) techniques allow for the delivery of high radiation doses to fields encompassing partial liver volumes as a component of combined modality cancer treatment. RT doses are limited by concerns for radiation-induced liver disease (RILD) [1]. Imaging changes may occur after treatment, in the absence of treatment-related toxicity, and must be distinguished from possible disease recurrence. In this report, we describe CT changes after chemoradiotherapy for cholangiocarcinoma as an example of the challenge of distinguishing treatment effect from disease recurrence.

## Case presentation

**Figure 1:**
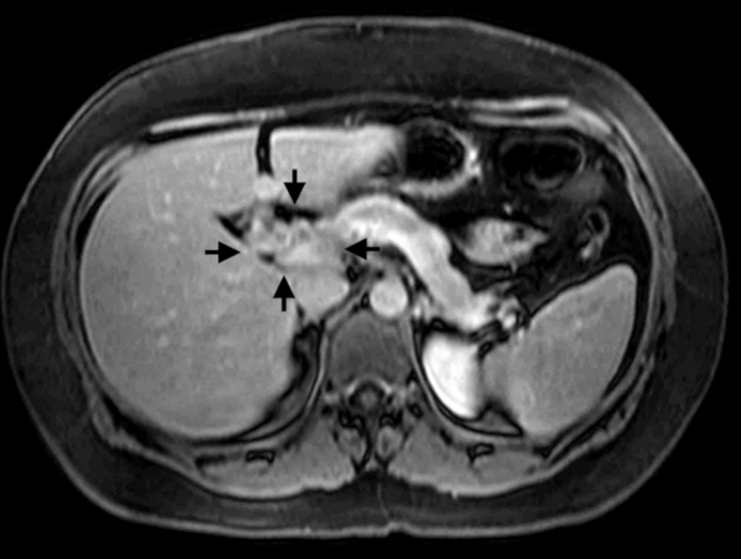
Axial T1, fat-suppressed, portal venous phase MRI performed prior to resection. Arrows define the boundaries of the cholangiocarcinoma.

A 40-year-old female presented with a one-month history of diffuse pruritus that failed to resolve with antihistamines. She developed right upper quadrant abdominal pain and jaundice. Contrast-enhanced magnetic resonance imaging (MRI) demonstrated a 2.1 × 1.6 cm porta hepatis mass involving the common bile duct (CBD) with ductal dilatation (Fig. 1). Endoscopic retrograde cholangiopancreatography revealed an irregular CBD with stricture of the proximal common hepatic duct at the level of the hepatic duct bifurcation. Biliary decompression was achieved through preoperative stent placement. Imaging studies revealed no evidence of regional lymphadenopathy or distant metastases.

**Figure 2:**
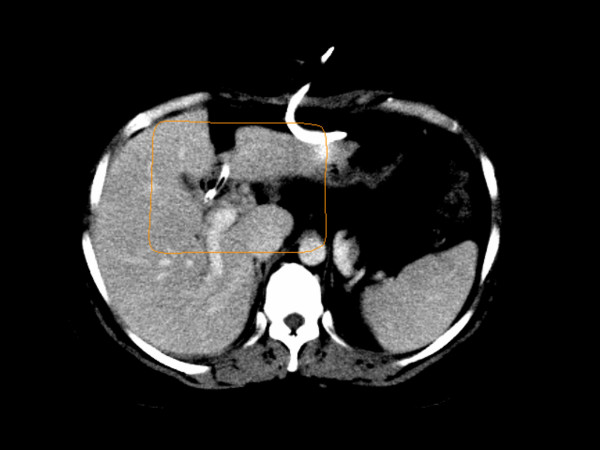
Axial image of CT scan performed with IV contrast for radiation treatment planning. The solid line marks the region that received at least 50.4 Gy during the radiotherapy course.

During exploratory laparotomy, palpation of the common bile duct revealed an obvious mass in the common hepatic duct region above the termination of the cystic duct. Neither hepatic dissemination nor regional lymphadenopathy was noted, and the surgeon proceeded to perform a cholecystectomy, choledochectomy, hepaticojejunostomy and reconstruction of the biliary enteric system. Adequate biliary drainage was achieved. Surgical pathology showed a poorly differentiated adenocarcinoma of the common bile duct, measuring 1.6 cm in greatest dimension. Resection margins were negative. Tumor cells infiltrated the fibromuscular layer of the hepatic duct with extension into the surrounding connective tissue, and all lymph nodes were negative. The patient was staged as IB (T2N0M0) cholangiocarcinoma, and was recommended to receive concurrent combined modality therapy consisting of capecitabine 1500 mg twice daily and external beam RT to a total dose of 50.4 Gy delivered in 28 daily fractions of 1.8 Gy. A CT was performed for RT treatment planning, and three-dimensional techniques were used to develop a four-field conformal plan. The 50.4 Gy isodose line displayed on the treatment planning CT (Fig. 2) represents the borders of the treated volume that received the prescribed dose.

**Figure 3:**
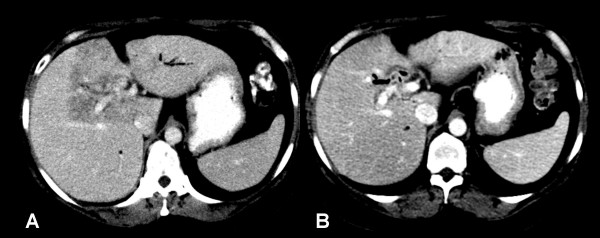
**(A) **Contrast-enhanced CT scan performed three months after completion of chemoradiotherapy. The arrows highlight the linear margins of the region of low attenuation. **(B) **CT obtained 9 months after radiation treatment. Oral and IV contrast were administered. The previously observed low attenuation region has resolved.

On routine follow-up three months after completing chemoradiation, the patient was asymptomatic and had returned to work full-time. Laboratory values were within normal limits. At this time, a CT scan of the abdomen and pelvis showed a low attenuation region in the liver with linear margins (Fig. 3A). The radiation oncologist was consulted to assist in evaluating the radiographic abnormality in order to distinguish post-radiation changes from local recurrence. When compared to the CT obtained for treatment planning (Fig. 2), the tomographic changes correspond closely to the radiation prescription isodose line. In order to verify that the imaging abnormality corresponded to the radiation portal, the radiation treatment planning CT and the follow-up diagnostic CT were co-registered using anatomical landmarks to confirm that the low-density region corresponded to the 50.4 Gy isodose line. Subsequently, the patient developed neither clinical hepatic toxicity nor disease recurrence. A CT scan obtained six months later (Fig. 3B) demonstrated nearly complete resolution of the previously observed post-RT hepatic parenchymal changes, with interval loss of hepatic volume and development of surface nodularity consistent with evolving post-RT changes with local fibrosis.

## Discussion

Conformal RT techniques may produce imaging changes that must be distinguished from disease recurrence. Although RT to the entire liver is known to produce clinical hepatic toxicity at doses of 30 to 35 Gy in standard fractionation, three-dimensional conformal planning techniques allow delivery of higher radiation doses to partial liver target volumes [1-3]. Current methods closely appose regions of high and low dose by escalating dose to the treatment volume and minimizing exposure to adjacent normal hepatic parenchyma [4]. Yamasaki et al. reviewed contrast-enhanced CT studies from 31 patients who received high-dose conformal radiation therapy to a partial liver volume and found minimal correlation between radiation-induced tomographic changes and clinical radiation hepatitis, which was observed in only 6% of subjects. 74% of patients studied displayed regions of low attenuation in treatment volume in post-treatment CT scans, but no scans showed linear margins [5]. The absence of crisp margins of attenuation may be explained by the use of nonaxial, noncoplanar overlapping fields in the study, in contrast to the four-field coplanar plan presented in this case. Other CT changes after partial volume hepatic radiation range from linear regions of low density corresponding to parallel opposed portals [6,7] to a peripheral, curved band of low attenuation in the upper liver after intensity-modulated radiation therapy for mesothelioma [8].

Imaging changes after chemoradiation of the liver are a separate entity distinct from RILD, a syndrome distinguished by the clinical triad of hepagatomegaly, ascites, and elevated liver enzymes, predominantly alkaline phosphatase. Although RILD may produce hepatic failure, most cases resolve within three months after radiation [4]. The pathologic features of RILD include severe central venous congestion of hepatic lobules, relative sparing of larger veins, and endothelial damage in small veins [4,9,10]. Clinically, RILD is similar to Budd-Chiari syndrome, but with sparing of larger veins such as the vena cava. Although RILD occurs with conventional radiation techniques [1-3], RT fields encompassing the whole liver may be safely delivered if the mean liver dose remains below 30 to 37 Gy [4,11,12]. Multiple three-dimensional models exist to predict tolerance of the liver to higher doses of partial volume radiation [4].

Imaging changes after partial volume hepatic radiation are usually subclinical and do not predict RILD [5]. In addition to CT changes involving the treated region after RT, other imaging findings include T1 hypointensity, T2 hyperintensity, and sustained contrast enhancement on MRI [13]; decreased echogenicity on ultrasound [14]; and decreased uptake of particulate reticuloendothelial contrast [15]. Collectively, imaging changes after hepatic RT may display the effects of edema or vascular congestion, consistent with a proposed vascular etiology for radiation-induced liver damage.

## Conclusion

Three-dimensional techniques for RT planning and delivery have expanded the possibilities for high-dose partial volume liver RT. Characteristic, transient radiographic changes occur in the absence of clinical RILD after hepatic RT and must be distinguished from recurrent disease on follow-up imaging studies. Because of the complexity of radiation treatment techniques, consultation across disciplines may be necessary to determine the significance of post-treatment imaging findings.

## Competing interests

The author(s) declare that they have no competing interests.

## Authors' contributions

TS participated in the design, coordinated the image analysis and drafted the manuscript. AO performed the image analysis and helped to draft the manuscript. FF participated in the study design and helped to draft the manuscript. PA participated in the study design and helped to draft the manuscript. ER participated in the study design and helped to draft the manuscript. AD conceived of the study, participated in the study design and helped to draft the manuscript. All authors read and approved the final manuscript.

## Consent

Written consent was obtained from the patient for publication of the report and accompanying imaging. A copy of the written consent is available for review by the Editor-in-Chief of this journal.

## References

[B1] Lawrence TS Robertson JM Anscher MS Jirtle RL Ensminger WD Fajardo LF Hepatic toxicity resulting from cancer treatment Int J Radiat Oncol Biol Phys 1995 31 1237 1248 7713785 10.1016/0360-3016(94)00418-K

[B2] Emami B Lyman J Brown A Coia L Goitein M Munzenrider JE Shank B Solin LJ Wesson M Tolerance of normal tissue to therapeutic irradiation Int J Radiat Oncol Biol Phys 1991 21 109 122 2032882 10.1016/0360-3016(91)90171-y

[B3] Wharton JT Delclos L Gallager S Smith JP Radiation hepatitis induced by abdominal irradiation with the cobalt 60 moving strip technique Am J Roentgenol Radium Ther Nucl Med 1973 117 73 80 4685535 10.2214/ajr.117.1.73

[B4] Dawson LA Ten Haken RK Partial volume tolerance of the liver to radiation Sem Radiat Oncol 2005 15 279 283 10.1016/j.semradonc.2005.04.005 16183482

[B5] Yamasaki SA Marn CS Francis IR Robertson JM Lawrence TS High-dose localized radiation therapy for treatment of hepatic malignant tumors: CT findings and their relation to radiation hepatitis AJR Am J Roentgenol 1995 165 79 84 7785638 10.2214/ajr.165.1.7785638

[B6] Jeffrey RB Jr Moss AA Quivey JM Federle MP Wara WM CT of radiation-induced hepatic injury AJR Am J Roentgenol 1980 135 445 448 6773363 10.2214/ajr.135.3.445

[B7] Willemart S Nicaise N Struyven J van Gansbeke D Acute radiation-induced hepatic injury: evaluation by triphasic contrast enhanced helical CT Br J Radiol 2000 73 544 546 10884753 10.1259/bjr.73.869.10884753

[B8] Munden RF Erasmus JJ Smythe WR Madewell JE Forster KM Stevens CW Radiation injury to the liver after intensity-modulated radiation therapy in patients with mesothelioma: an unusual CT appearance AJR Am J Roentgenol 2005 184 1091 1095 15788578 10.2214/ajr.184.4.01841091

[B9] Reed GB Cox AJ The human liver after radiation injury Am J Pathol 1966 48 597 611 5327788 PMC1916442

[B10] Ogata K Hizawa K Yoshida M Kitamuro T Akagi G Kagawa K Fukuda F Hepatic injury following irradiation – a morphologic study Tokushima J Exp Med 1963 9 240 251 14049847

[B11] Lawrence TS Ten Haken RK Kessler ML Robertson JM Lyman JT Lavigne ML Brown MB DuRoss DJ Andrews JC Ensminger WD Lichter AS The use of 3-D dose volume analysis to predict radiation hepatitis Int J Radiat Oncol Biol Phys 1992 23 781 788 1618671 10.1016/0360-3016(92)90651-w

[B12] Dawson LA Normolle D Balter JM McGinn CJ Lawrence TS Ten Haken RK Analysis of radiation-induced liver disease using the Lyman NTCP model Int J Radiat Oncol Biol Phys 2002 53 810 821 12095546 10.1016/s0360-3016(02)02846-8

[B13] Onaya H Itai Y Ahmadi T Yoshioka H Okumura T Akine Y Tsuji H Tsujii H Recurrent hepatocellular carcinoma versus radiation-induced hepatic injury: differential diagnosis with MR imaging Magn Reson Imaging 2001 19 41 46 10.1016/S0730-725X(01)00218-1 11295345

[B14] Garra BS Shawker TH Chang R Kaplan K White RD The ultrasound appearance of radiation-induced hepatic injury: correlation with computed tomography and magnetic resonanace imaging J Ultrasound Med 1988 7 605 609 3062187 10.7863/jum.1988.7.11.605

[B15] Padhani AR Husband JE Gueret Wardle D Radiation induced liver injury detected by particulate reticuloendothelial contrast agent Br J Radiol 1998 71 1089 1092 10211072 10.1259/bjr.71.850.10211072

